# Transgenic Citrus Expressing an *Arabidopsis* NPR1 Gene Exhibit Enhanced Resistance against Huanglongbing (HLB; Citrus Greening)

**DOI:** 10.1371/journal.pone.0137134

**Published:** 2015-09-23

**Authors:** Manjul Dutt, Gary Barthe, Michael Irey, Jude Grosser

**Affiliations:** 1 Citrus Research and Education Center, University of Florida, Lake Alfred, Florida, United States of America; 2 Southern Gardens Citrus, Clewiston, Florida, United States of America; University of Tsukuba, JAPAN

## Abstract

Commercial sweet orange cultivars lack resistance to Huanglongbing (HLB), a serious phloem limited bacterial disease that is usually fatal. In order to develop sustained disease resistance to HLB, transgenic sweet orange cultivars ‘Hamlin’ and ‘Valencia’ expressing an *Arabidopsis thaliana NPR1* gene under the control of a constitutive CaMV 35S promoter or a phloem specific *Arabidopsis* SUC2 (*AtSUC2*) promoter were produced. Overexpression of *AtNPR1* resulted in trees with normal phenotypes that exhibited enhanced resistance to HLB. Phloem specific expression of NPR1 was equally effective for enhancing disease resistance. Transgenic trees exhibited reduced diseased severity and a few lines remained disease-free even after 36 months of planting in a high-disease pressure field site. Expression of the *NPR1* gene induced expression of several native genes involved in the plant defense signaling pathways. The *AtNPR1* gene being plant derived can serve as a component for the development of an all plant T-DNA derived consumer friendly GM tree.

## Introduction

In the United States, Huanglongbing (HLB) is a serious disease of citrus associated with the phloem limited bacterium *Candidatus* Liberibacter asiaticus (*C*Las) [1]. This disease is spread by the Asian citrus psyllid (ACP) vector, *Diaphorina citri* (Kuwayama) [2]. HLB was first detected in the United States in August 2005 and since then has rapidly moved into several citrus producing areas [3, 4]. Tree decline is usually preceded by a decline in the quality of the fruit and fruit drop. Fruit from infected trees are smaller, yield less juice, have higher acidity and lower sugar and peel color than fruits from uninfected trees [5]. Infected citrus trees will irrevocably decline. Currently, HLB management consists of preventing trees from becoming infected [4]. Prevention includes protection of the young flush from the psyllid vector [6] or destruction of infected plant material to prevent further spread of the disease. Due to the lack of rapid curative methods to control HLB, prevention of new infections is essential in HLB management [7]. New infections could be prevented and the disease could be managed by planting trees that are tolerant or resistant to the disease [6].

Utilization of resistant germplasm to slow the spread of HLB in citrus is difficult due to the lack of commercially available resistant rootstock/scion combinations that can provide a robust protection and prevent infection. Identification and incorporation of resistance traits from tolerant *Citrus* spp. and *Citrus* relatives is a potential disease management strategy [8]. However, citrus crop improvement using conventional breeding methods is difficult and time consuming due to the long juvenile phase in citrus, which can vary from 4 to 12 years [9]. Genetic improvement of citrus through genetic engineering remains the fastest method for improvement of an existing citrus cultivars and has been a key component in the University of Florida’s genetic improvement strategy [10].

**Fig 1:**
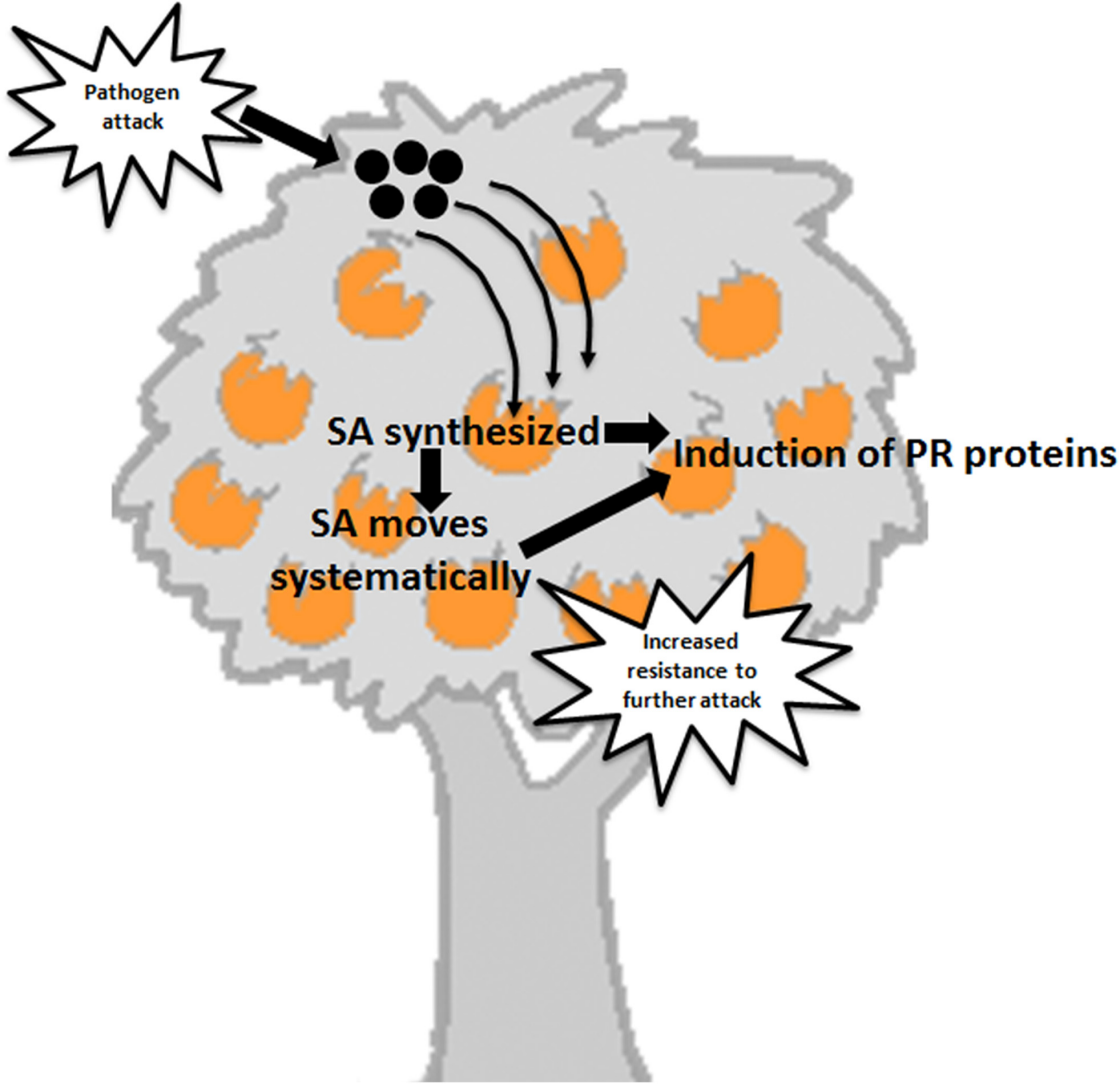
The process of systemic acquired resistance (SAR) induction in citrus.

Genetic improvement of citrus using genes that allow plants to defend themselves against pathogens utilizing systemic acquired resistance (SAR) has resulted in the production of transgenic canker resistant trees [11]. SAR is a plant defense response resulting in the expression of specific defense genes that enhances innate resistance to further infection by pathogens [12]. Utilization of SAR is a novel method to employ the plant’s inherent immune system to reduce disease development and spread (Fig 1). SAR provides protection against a broad spectrum of microorganisms and is associated with the production of pathogenesis-related (PR) proteins [13]. This defense response is induced by salicylic acid (SA) [14], since plants that fail to produce salicylic acid also fail to develop SAR, nor do they express pathogenesis-related (PR) genes in the uninoculated leaves [15]. These plants are also more susceptible to pathogen infection [16]. For example transgenic *Arabidopsis* plants overexpressing the *nahG* gene encoding the SA hydroxylase enzyme are unable to accumulate SA due to its degradation by the SA hydroxylase enzyme into catechol. Such plants are very susceptible to infection by *Pseudomonas syringae* and *Peronospora parasitica*. Several *Arabidopsis* mutants which are salicylic acid induction-deficient are unable to accumulate SA after pathogen inoculation and are very susceptible to pathogens [17].

Non-expressor of Pathogenesis Related genes 1 (*NPR1*) gene is a key regulator in the signal transduction pathway that leads to SAR since the *npr1* mutant in *Arabidopsis* fails to respond to various SAR-inducing agents and exhibits very low expression of several PR genes. The NPR1 gene may act as a regulator of the transcription factor/s that controls PR gene expression [18] and mediates the salicylic acid induced expression of PR genes and SAR [19]. Plants over expressing *NPR1* exhibit enhanced resistance to several pathogens [20].

We have produced and evaluated several transgenic sweet orange trees overexpressing *AtNPR1* either in the phloem tissues (where HLB resides) via utilization of a phloem specific *Arabidopsis* sucrose-proton symporter 2 (*AtSUC2*) promoter or a constitutive CaMV 35S promoter for HLB resistance. Evaluation of these transgenic plants demonstrates that overexpressing the *AtNPR1* can result in effective HLB resistance in citrus.

## Materials and Methods

### Development of plant transformation vectors

**Fig 2:**
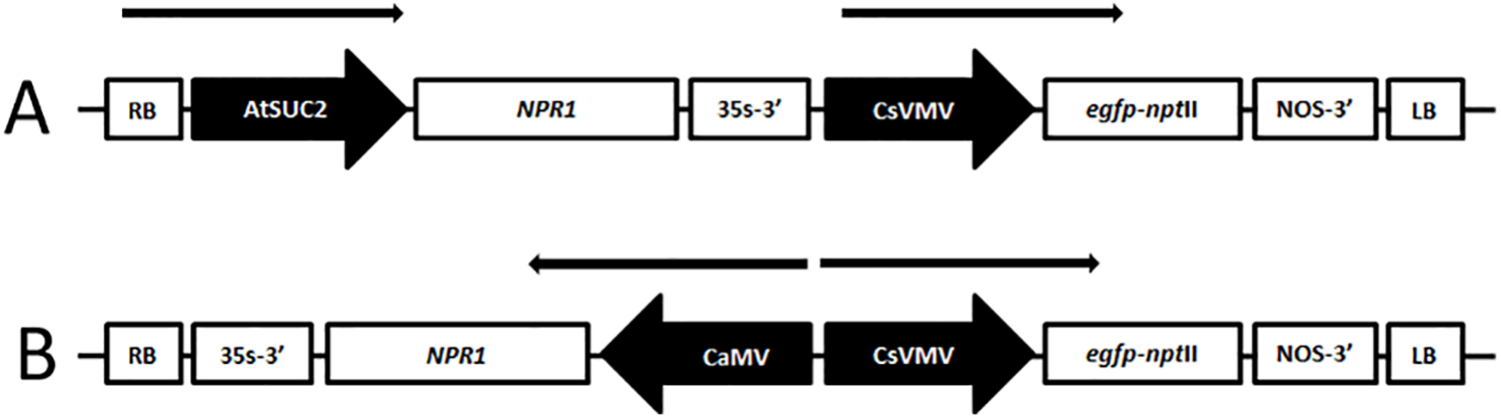
Schematic representation of T-DNA region of the binary vectors used in this study. A) Phloem targeted gene construct B) Constitutive expression gene construct. CaMV, double enhanced (2 x -343 to -90) CaMV 35S promoter; dCsVMV, double enhanced (2 x -443 to -123) CsVMV promoter; AtSUC2, The *Arabidopsis* sucrose synthase promoter, *egfp*/*npt*II, bifunctional enhanced green fluorescent protein and neomycin phosphotransferase II fusion gene; NOS-3’, termination site and polyadenylation signal of the NOS transcript; 35S-3’, termination site and polyadenylation signal of the CaMV 35S transcript; RB, right border; LB, left border. The arrow represents the direction of transcription.

The cDNA sequence of *AtNPR1* (U76707) is available in the NCBI database. Primers to amplify the *AtNPR1* were designed using the bioinformatics software Vector NTI^**®**^ (Life Technologies, NY, USA) to incorporate a *Bam*HI restriction site at the 5’ end and a *Not*I site at the 3’ end. Total RNA was isolated from 100 mg of tissue from young, fully expanded leaves of *Arabidopsis thaliana* cv. Columbia using a RNeasy Mini Kit (Qiagen Inc., Valencia, CA). cDNA was synthesized from 500 ng total RNA using Oligo (dT) primer and a RETROscript® RT-PCR kit as described by the manufacturer (Applied Biosystems, Austin, TX). The cDNA product was used as a template for PCR using primers as described above. The gene was excised as a *Bam*HI/*Not*I fragment and inserted between a double enhanced CaMV 35S promoter (d35S) and a CaMV 35S terminator (3’CaMV) of a pUC18-derived plasmid pDR. Variations of this cloning vector containing the phloem specific *Arabidopsis* SUC2 promoter (NCBI accession: JQ733913) were also produced. A *Hin*dIII DNA fragment containing the expression cassette d35S (or AtSUC2)–NPR1 gene—3’CaMV were isolated and cloned into the unique *Hin*dIII site of a pBIN19-derived binary vector. This vector, containing a bifunctional *npt*II/*egfp* fusion gene has been described earlier [21]. All constructions were verified first by restriction analysis and then by DNA sequencing (Fig 2). Each vector was introduced into *A*. *tumefaciens* strain EHA105 [22] by the freeze-thaw method [23].

### Transformation, selection and propagation of regenerants

*Agrobacterium* mediated transformation of etiolated sweet orange epicotyl segments from the cultivars ‘Hamlin’ and ‘Valencia’ were carried out as described previously [24]. EGFP-specific fluorescence in putative transgenic lines was evaluated using a Zeiss SV11 epi-fluorescence stereomicroscope equipped with a light source consisting of a 100W mercury bulb and a FITC/GFP filter set with a 480 nm excitation filter and a 515 nm longpass emission filter producing a blue light (Chroma Technology Corp., VT, USA). Transgenic ‘Hamlin’ and ‘Valencia’ sweet orange shoots are very difficult to root *in vitro* [24] and in this study no attempt was made to root any of our EGFP expressing transgenic lines. Instead, EGFP positive transgenic shoots were micrografted *in vitro* onto 1 month old Carrizo citrange (*Citrus sinensis* (L.) Osbeck × *Poncirus trifoliata* (L.) Raf.) nucellar rootstock seedlings. After a month of growth *in vitro*, the grafted shoots were potted into a peat based commercial potting medium (Metromix 500, Sun Gro Horticulture, Bellevue, WA) and acclimated under greenhouse conditions. An *ex vitro* micrografting technique was subsequently used to rapidly propagate transgenic plants onto 6 month old Carrizo rootstocks [25]. Plants were grown for an additional 9 to 12 months before evaluation of disease resistance. Transgenic lines with the AtSUC2-NPR1 construct had an ‘A’ added in as a suffix.

### Molecular analysis of transformants

Citrus genomic DNA, was isolated from 100 mg of young transgenic leaf tissues using the GenElute™ Plant Genomic DNA Miniprep Kit (Sigma-Aldrich Corp., St. Louis, MO). Duplex PCR was carried out in a thermal cycler (MJ Research, Watertown, MA) using GoTaq® Green Master PCR Mix (Promega Corp, Madison WI) and appropriate primers (NP51, 5’ ATG GAC ACC ACC ATT GAT GGA TTC 3’ and NP32, 5’ ACG ACG ATG AGA GAG TTT ACG GTT AG 3’) and (EG-51, 5’ATG GTG AGC AAG GGC GAG GAG CTG T3’ and EG-32, 5’CTT GTA CAG CTC GTC CAT GCC GAG A3’) to confirm the presence of the *AtNPR1* and *egfp* transgenes respectively in the genome of transgenic citrus plants. Amplified DNA fragments were electrophoresed on a 1% agarose gel and visualized under UV light. All images were recorded and digitized. All samples for the detection of *C*las in transgenic citrus were analyzed by qPCR at the diagnostic laboratory of Southern Gardens Citrus in Clewiston, FL, USA. Four to five fully expanded and hardened leaves were collected from dark green colored branches. Leaves were placed in a zip lock plastic bag, kept cool and out of direct sunlight and subsequently shipped by overnight mail and processed the following day. DNA was isolated from 100 mg of leaf petiole tissue using BioSprint DNA Plant kits (Qiagen, Valencia, CA) on a BioSprint 96 instrument (Qiagen, Valencia, CA). DNA was dissolved in 100 μl of TE buffer and 2 μl were used for qPCR. qPCR was performed in a 25ul volume on an ABI7300 (Life Technologies, Grand Island, NY) using TaqMan Universal PCR Master Mix (Life Technologies, Grand Island, NY) using the Li primers [26].

Southern blot analysis was carried out for confirmation of copy number in selected transgenic citrus plants. Fifteen μg of *Eco*RI digested genomic DNA immobilized on a positively-charged nylon membrane was probed with a DIG-labeled *AtNPR1* probe. Following hybridization to the probe, the chemiluminescence substrate CDP-Star was used for immunological detection of hybridization signals using X-ray film autography. Validation of transgene copy number was carried out using qPCR essentially as described previously [27].

RNA was isolated from 100 mg of leaf tissue using an RNeasy Mini Kit (Qiagen Inc. Valencia, CA) according to the manufacturer’s protocol. Real-time quantitative PCR (RT-qPCR) was performed as outlined before with minor modifications [28]. The RT-qPCR reactions were performed with a final volume of 25 μl using the TaqMan^®^ RNA-to-Ct^TM^ one-step kit (Applied Biosystems, Foster City, CA) according to the manufacturer’s instructions. The one-step kit parameters consisted of 20 min incubation at 48°C followed by 10 min incubation at 95°C and 40 cycles at 95°C for 15 s and 60°C for 1 min. Each qPCR contained negative and non-template/water controls in addition to the sample being tested. Experiments were repeated at least twice with three replicates and the data was analyzed using Applied Biosystems software version 2.0.6. Relative quantitation was measured using the comparative Cq method (2^-ΔΔCt^). The fold change in the relative expression was then determined by calculating 2^-ΔΔCt^ [29]. The sequences of the primers and probes including the reporter fluorescent dye and dark quencher dye used in the RT-qPCR are shown in Table 1. The 18S rRNA housekeeping gene was used as an endogenous control. A set of reaction mixtures composed of one to five copies of *AtNPR1* gene equivalences, was used to establish a standard curve for the evaluation of transgene copy number. The method as outlined earlier was used to prepare the set of reaction mixtures [30].

**Table 1:** Primers used in real-time PCR assay of transgenic citrus plants.

Target gene	Amplicon length (bp)	Primer/probe sequence 5’→ 3’ ^A^
AtNPR1	113 bp	TGCATCAGAAGCAACTTTGG
		6FAM-CGCAAAACAAGCCACTATG
		GGCCTTTGAGAGAATGCTTG
CsPR1	88 bp	AACTCGCCTCAAGACTACCT
		6FAM-TCACAATTCAGCTCGAGCAGCAGTC
		TGCAACTGTGTCGTTCCATA
CsPR2	92 bp	ACTTCGCTCAGTACCTTGTTC
		6FAM-ATCAACAGAGCCGGCCTTGGAAA
		GGCAGTGGAAACCTTGATTTG
CsWRKY70	106 bp	CTGTGCTCGGTACTACTGTTAC
		6FAM-TGAGAAGTATCAGCAGCAGCAGGC
		CGGCGATAGTCATCGGAATTA
18S rRNA	112 bp	TCGGGTGTTTTCACGTCTCA
		HEX-TGGAACTCTTGGATTTTGCC
		CGCCGTAGGTGAGGTAGC

^A^ Primer/probe sequences listed in the column include the forward primer in the first line, followed by the probe sequence in the second and the reverse primer sequence in the third line.

### HLB resistance studies

**Fig 3:**
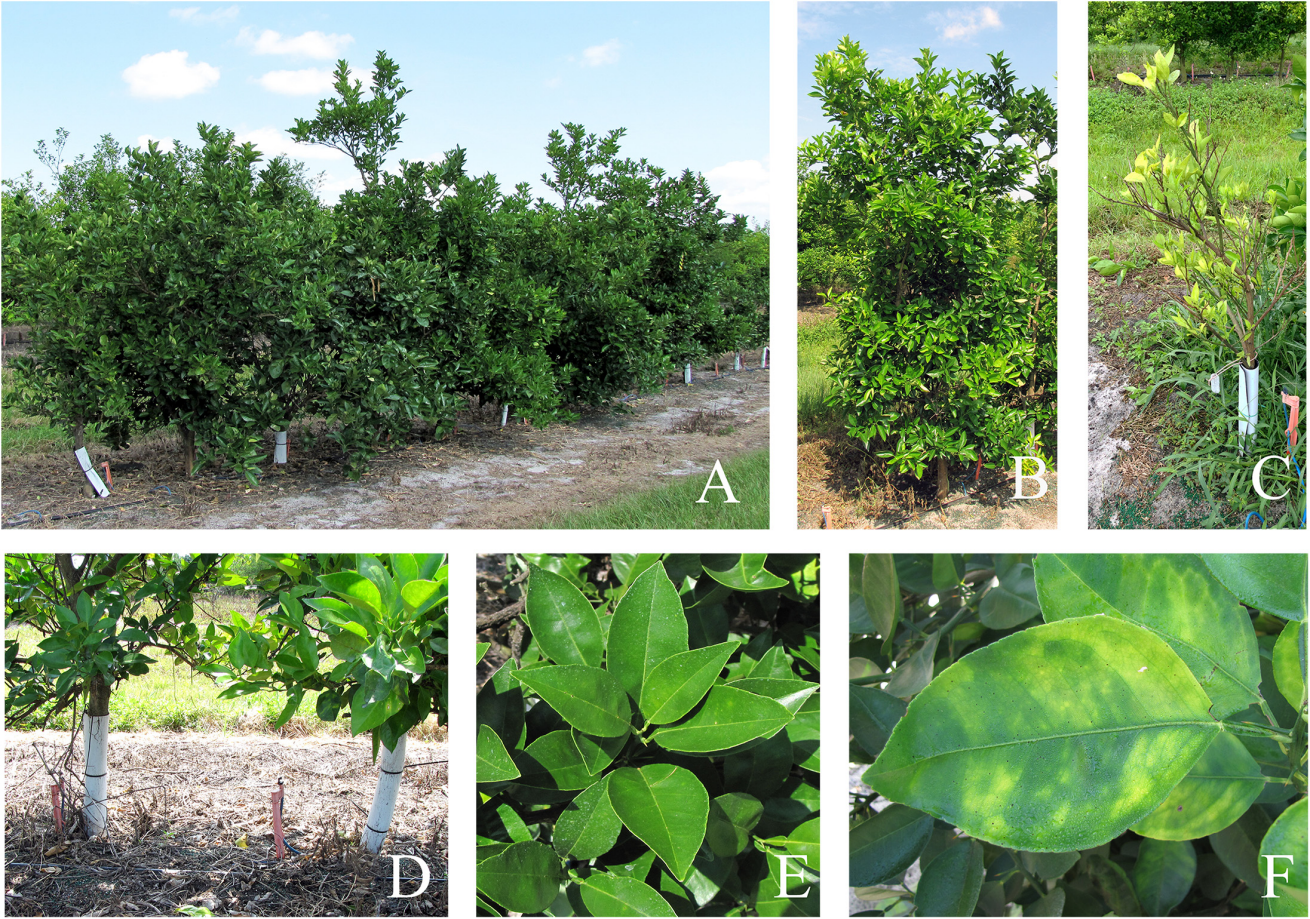
A) A set of transgenic trees with the *AtNPR1* construct, B) Close-up of an HLB positive transgenic tree with the *AtNPR1* construct, C) A heavily infected HLB positive control tree, D) 2 feet spacing between two adjacent trees in our field plot. Normal citrus trees are usually planted at an 8 feet spacing or more, E) Close-up of a healthy flush, F) Close-up of an HLB infected leaf.

Disease resistance to HLB in this study was evaluated in two ways. First, in a no-choice greenhouse evaluation study, 3 replicated clones of independent transgenic plant lines were exposed to free flying *C*Las positive ACP continuously for two years. Trees were routinely pruned and fertilized with both 9 month slow release and liquid fertilizer to stimulate new flush production. These trees were evaluated every 6 months for two years for the presence of HLB by qPCR as outlined before. ACP were also randomly evaluated during this study for the presence of the *C*Las. In the second concurrent study, selected transgenic trees and controls (consisting of 10% of the total tree population) were planted in a high disease pressure (over 90% infection rate) field site in a randomized block design experiment. In our test site, trees were planted at a narrow spacing of 2 feet to maximize land utilization (Fig 3D). These trees were similarly evaluated every 6 months for three years by qPCR for the presence of HLB. Data were analyzed to calculate standard error using MS Excel.

## Results

### Production of genetically modified citrus plants

A total of 36 transgenic ‘Hamlin’ and ‘Valencia’ sweet orange lines expressing the 35S-NPR1 construct and 22 lines with the AtSUC2-NPR1 construct were regenerated. Transgenic plants were selected based on visual selection using EGFP fluorescence. A 20% transformation efficiency was observed using ‘Hamlin’ epicotyl segments while the transformation efficiency using ‘Valencia’ epicotyl segments were significantly lower (3%). These shoots were micrografted onto Carrizo seedlings to expedite plant growth. *In vitro* micro grafted shoots were hardened after 4–6 weeks of grafting and transferred to a greenhouse, After 4 months of growth, plants were analyzed for the presence of the *AtNPR1* gene before being micro grafted *ex vitro* [25]. We did not observe major phenotypic abnormalities in a majority of the transgenic plants regenerated in this study. Two lines obtained with the 35S-NPR1 construct exhibited abnormally slow growth and were deemed unsuitable and discarded.

### Verification of transgene integration, transcript accumulation and transgene copy

**Fig 4:**
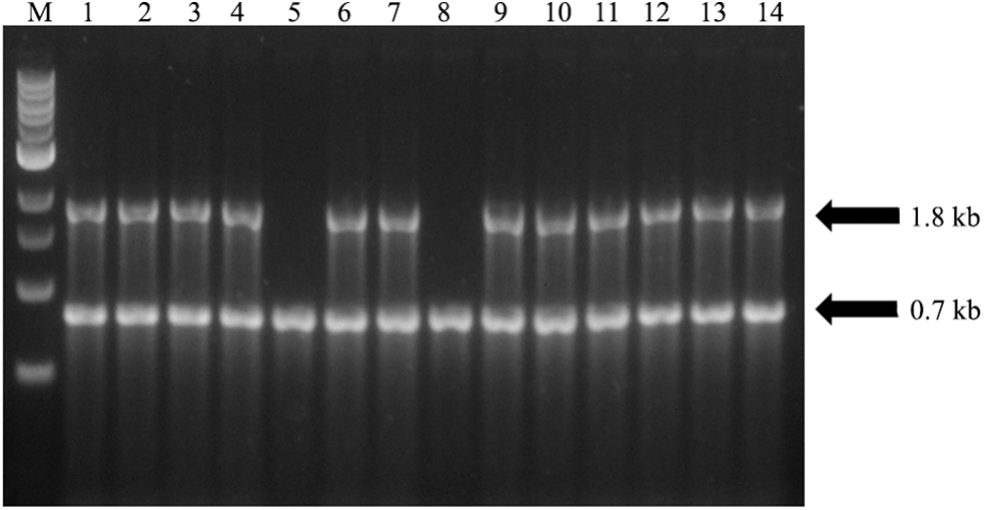
Duplex PCR amplification products of the *AtNPR1*and *egfp* genes from genomic DNA of transgenic sweet orange citrus plants. Transgenic lines 1 to 7 are 35S-NPR1 lines while lines 8 to 14 are AtSUC2-NPR1 lines. Amplification was carried out using gene specific primers which gave the expected 1.8 kb *AtNPR1* fragment and 0.7 *egfp* fragment (arrows). M, 1kb DNA ladder; 1–14 are 14 randomly selected individual transgenic lines.

**Fig 5:**
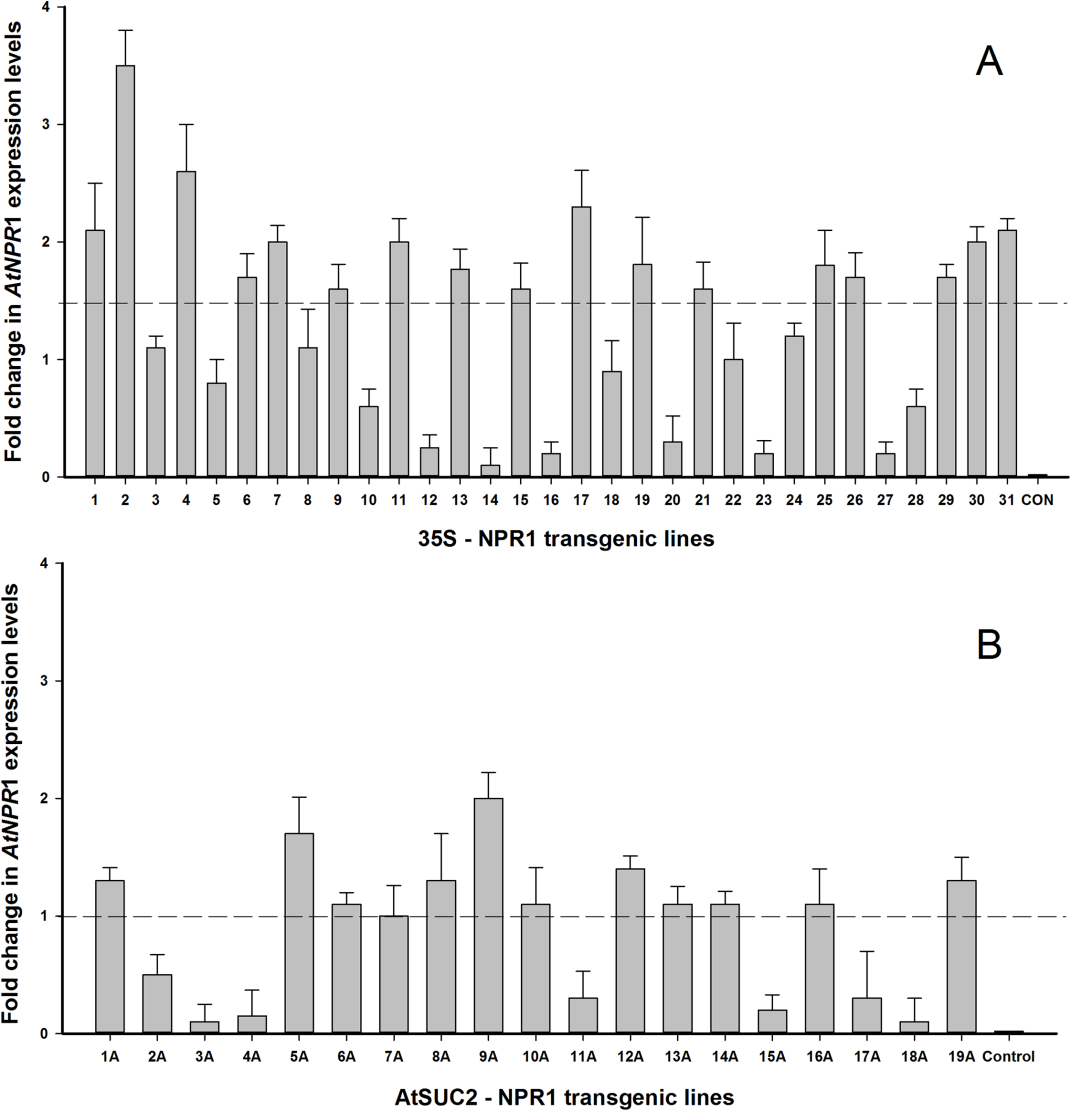
Quantification of *AtNPR1* activity using qPCR. Total RNA extracted from entire sweet orange leaf (A) or specifically midrib and petioles (B) was used as template. Sequence of primers used to amplify the *AtNPR1* gene is detailed in Table 1. Transgenic lines 1 to 16 containing the 35S-NPR1 cassette are ‘Hamlin’ while lines 17 to 31 are ‘Valencia’. Transgenic lines 1 to 12 containing the AtSUC2-NPR1 cassette are ‘Hamlin’ while lines 13 to 19 are ‘Valencia’. Three independent clones were tested from each transgenic line. Total RNA from a non-transgenic plant was also included to verify the accuracy of the amplification process. Transgenic plants that had a level of expression greater than indicated by the dotted line were considered to exhibit a relative high level of expression of *AtNPR1*.

Thirty one transgenic Hamlin’ and ‘Valencia’ sweet orange lines expressing the 35S-NPR1 construct had both the *egfp* as well as the *AtNPR1* genes incorporated into the genome as confirmed by PCR. Nineteen lines with the AtSUC2-NPR1 construct behaved similarly. Results from 14 arbitrarily selected samples are shown in Fig 4. Lines without the *AtNPR1* gene were discarded and the rest analyzed for mRNA production through qRT-PCR (Fig 5). Transgenic lines with the 35S-NPR1 construct that had a 1.5 fold higher level of expression were considered to exhibit a relative high level of expression of *AtNPR1*. In a similar manner, transgenic lines with the weaker phloem specific AtSUC2-NPR1 construct that had a 1 fold higher level of expression were considered to exhibit a relative high level of expression of *AtNPR1*. We obtained 17 constitutively expressing lines (35S-NPR1) and 12 phloem specific lines (AtSUC2-NPR1) that were considered to have a relative high level of expression of *AtNPR1* (Fig 5; dotted line). The transgenic lines were subsequently micrografted *ex vitro* to produce a population of trees for disease resistance analyses. Based on our greenhouse and field results, transgenic lines could be categorized as asymptomatic and resistant, symptomatic but tolerant or susceptible to HLB. We selected the lines 2, 4, 9A, 11A and 18 as representatives of our transgenic population for detailed molecular analyses.

**Fig 6:**
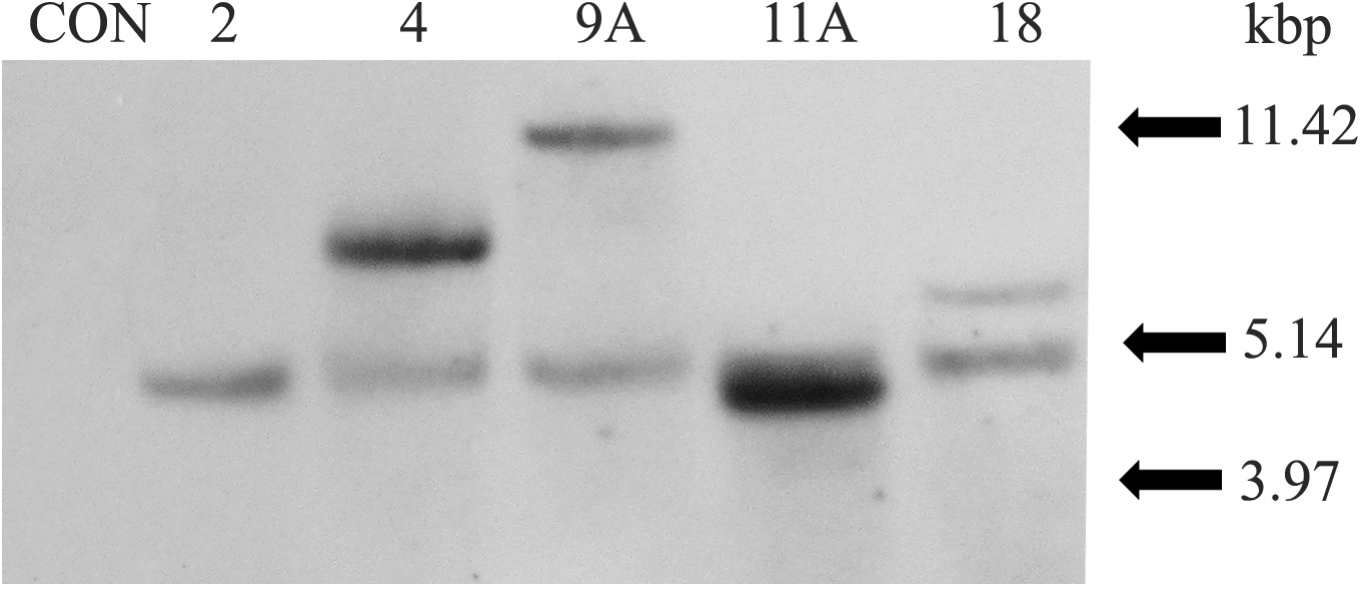
Southern hybridization analysis of total DNA from leaf tissue of five *AtNPR1* transformed sweet orange lines (2, 4, 9A, 11A, 18) and a non-transgenic control plant. Lines denoted with a number are constitutively expressing lines while lines with the suffix ‘A’ are phloem specific lines.

Southern blot hybridization was used to determine the number of inserted *AtNPR1* copies in the genomes of the selected transgenic lines. All transgenic lines demonstrated *AtNPR1* integration profiles whereas none was detected from the control plant (Fig 6). The transgenic line, 2, had one gene copy integrated into the genome. Transgenic lines 2, 4, 9A and 11A are ‘Hamlin’ while the line 18 is a ‘Valencia’ sweet orange. Transgenic plant lines No. 4, 9A and 18 had 2 copies while based on the intensity of the band we predicted that 11A line had 2 to 3 copies. These results were confirmed by q-PCR (Table 2). No amplification was detected from a non-transformed control plant (data not shown).

**Table 2:** Transgene copy number determination using quantitative real-time PCR by comparison of transgenic lines with external plasmid controls.

Transgenic Line	Cultivar	Mean Cp ^A^	STD Cp ^B^	Mean Conc. ^C^ ^,^ ^D^
NPR1-2	Hamlin	25.550	0.115	1.101
NPR1-4	Hamlin	25.160	1.378	1.605
NPR1-9A	Hamlin	25.064	0.639	1.738
NPR1-11A	Hamlin	24.412	0.867	2.643
NPR1-18	Valencia	24.738	0.184	2.190
Plasmid-1C^E^	-	25.727	0.132	0.818
Plasmid-2C	-	24.746	0.079	2.180
Plasmid-3C	-	24.022	0.149	3.185
Plasmid-4C	-	23.567	0.115	3.816
Plasmid-5C	-	23.107	0.232	4.753

^A^ Average values of crossing point from three sample replicates.

^B^ Standard deviations.

^C^ Average values of extrapolated concentration relative to a single transgene copy.

^D^ Copy number.

^E^ Plasmid DNA used for copy number calculations

### PR gene expression

**Fig 7:**
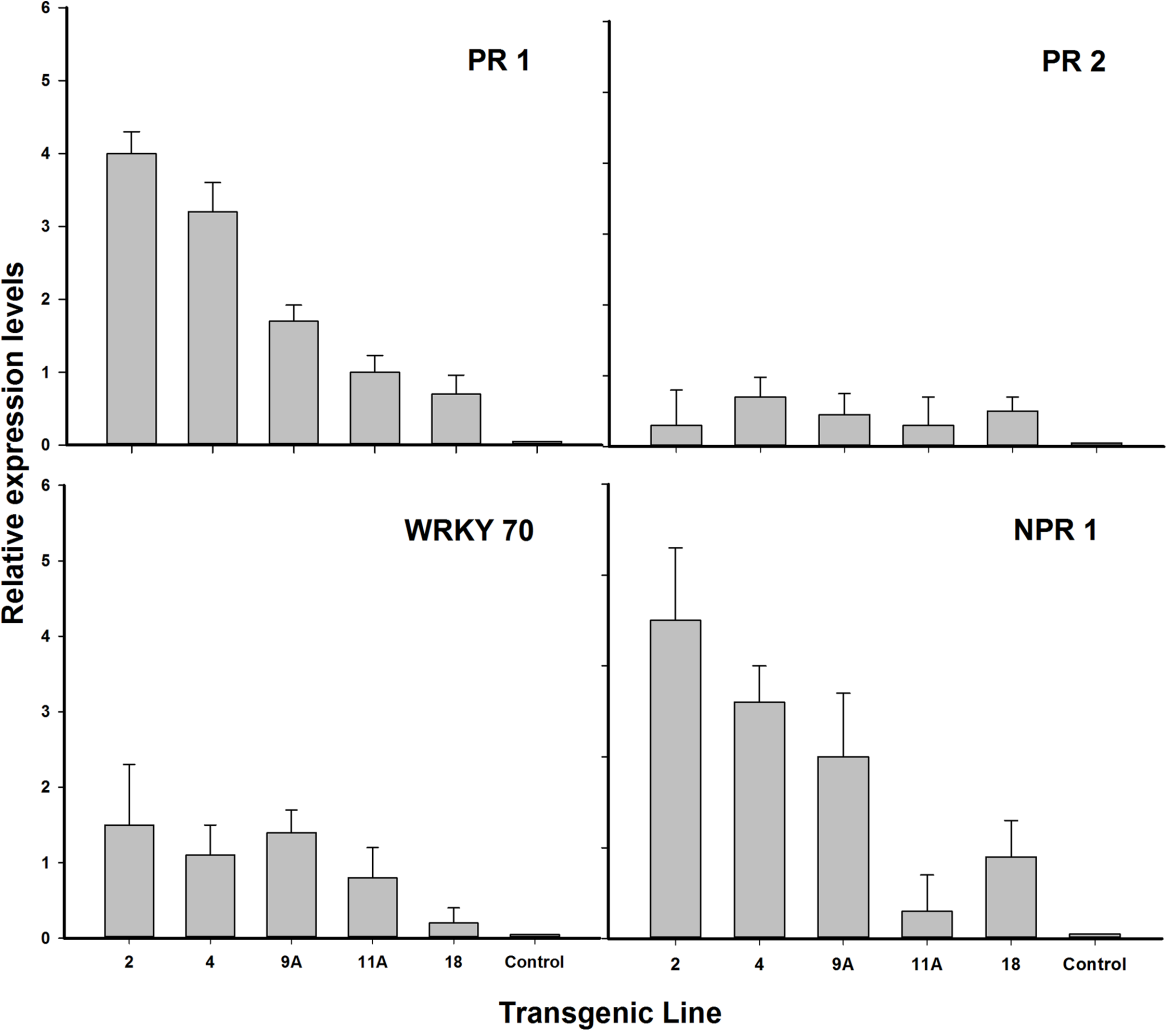
Quantification of gene activity using qPCR. Sequence of primers used in the qPCR process is detailed in Table 1. Three independent clones were tested from each transgenic line. Total RNA from a non-transgenic plant was also included to verify the accuracy of the amplification process. Selected data from Fig 4 (*AtNPR1*) is included here for comparison.

The selected transgenic lines that were analyzed for copy number by Southern blot hybridization were also evaluated for PR gene expression using qRT-PCR. The pathogenesis-related *PR1* gene is induced by *NPR1* [31] and significant variation in *PR1* gene expression was observed in these transgenic lines (Fig 7). All tested lines had enhanced PR1 gene expression. Transgenic line 2 exhibited a fourfold level in *PR1* gene expression compared to the control. Lower expression levels were observed in the other lines. Expression levels of the *PR2* gene, a SAR marker gene in citrus were also higher in all transgenic lines evaluated. However gene expression was less than 1 fold higher in all lines analyzed and there was no statistical significance between the evaluated transgenic lines. *WRKY70* is a direct target for *NPR1* and plays a role as a positive regulator of SA-mediated gene expression and resistance [32]. WRKY70 expression was not observed to be significantly different in any *NPR1* overexpressing transgenic line except in line 18. The *AtNPR1* expression levels in the transgenic lines were many fold higher than that observed in the non-transformed control plant, except for transgenic line 11A (Figs 5 and 7).

### Susceptibility of transformed lines to Huanglongbing

**Fig 8:**
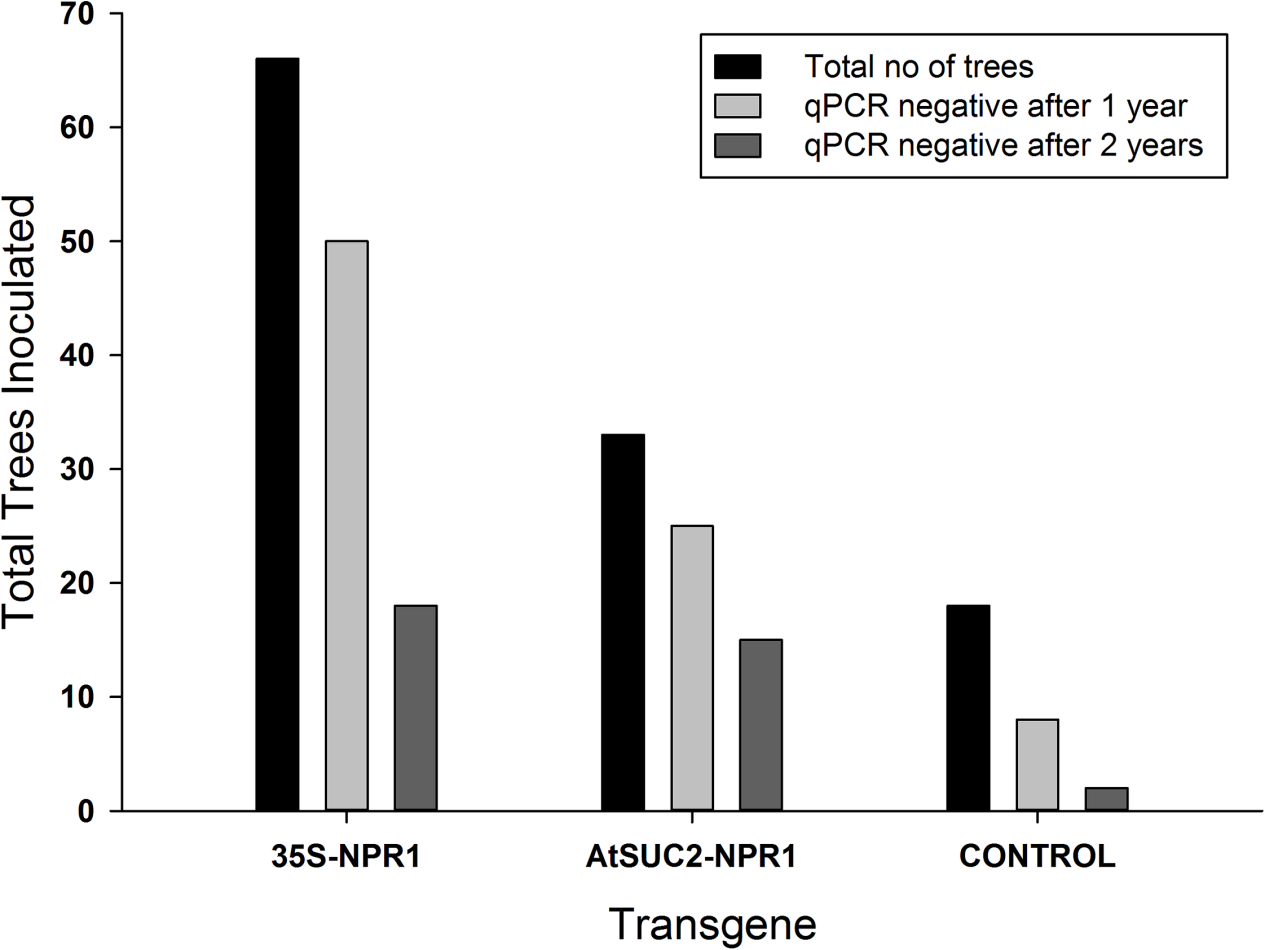
Survival of transgenic plants and control after 1 and 2 years in a no-choice greenhouse evaluation study and exposed to free flying potentially *C*Las containing psyllids.

Huanglongbing is caused by the phloem-limited, fastidious α-proteobacteria *C*Las spp. A majority of the trees tested positive for the bacterium in the second year of evaluation. Approximately 45% of the trees expressing *AtNPR1* under the control of the phloem specific promoter were HLB negative while 27% of the trees expressing *AtNPR1* under the control of the constitutive 35S promoter remained HLB negative (Fig 8). We did not detect the bacterium in transgenic lines 2, 4 and 9A for the duration of this study. Transgenic line 11A tested positive within 6 month and the severely infected trees were discarded after 18 months of infection (Table 3). Control trees tested positive for the presence of the *C*Las within 6 months after infection and remained positive for the entire duration of the study. In the second study, trees were planted in a high disease pressure field site. The results from that study are presented in Table 4. Transgenic line 2 remained *C*Las free for the duration of the study except for the 24 month sampling period when it tested positive. Line 4 tested positive at the 30 month sampling period while line 9A tested positive at 30 months but was *C*Las free at 36 months. Both of these lines did not decline in health and showed continued growth with periodic flushes. Line 11A tested positive after 18 months in the field and remained *C*Las + for the duration of the test period. The tolerant lines 2 and 9A also did not demonstrate any visual symptoms for the duration of the study while line 4 developed symptoms, tested positive for *C*Las but continued growth at a similar rate to the lines 2 and 9A. Transgenic line 18 was the most susceptible line and tested positive for *C*Las within 6 months after planting. Trees from this line began dying after 30 months in the field and were all dead within 36 months of planting in the field. Similar results were observed in the non-transgenic control trees (Fig 3).

**Table 3:** Quantification of *C*Las bacterial titers following qPCR from leaf petiole and midribs of the transgenic plants and controls grown under greenhouse conditions and exposed to free flying, potentially CLas positive psyllids. The mean threshold cycle values (Ct) at specified time intervals are demonstrated.

	Transgenic line #2	Transgenic line #4	Transgenic line #9A	Transgenic line #11A	Transgenic line #18	Control
6 months	-	-	-	29.29± 2.3	NT	23.48±1
12 months	-	-	-	23.63±1.4	NT	22.04±2.2
18 months	-	-	-	21.19±3.1	NT	20.78±4.5
24 months	-	-	-	*	NT	*

*; dead trees, NT; not tested, Standard errors were calculated from three replicates.

**Table 4:** Quantification of *C*Las bacterial titers following qPCR from leaf petiole and midribs of the transgenic plants and controls grown under field conditions in a high disease pressure test site. The mean threshold cycle values (Ct) at specified time intervals are demonstrated.

	Transgenic line #2	Transgenic line #4	Transgenic line #9A	Transgenic line #11A	Transgenic line #18	Control
6 months	-	-	-	-	37.42±3.1	38.39±3.3
12 months	-	-	-	-	30.13±2.5	26.20±1.8
18 months	-	-	-	33.81±4.1	23.81±4.1	27.69±1.5
24 months	36.00^a^	-	-	27.72±2.3	29.02±1.6	22.87±1.6
30 months	-	33.02±2.4	38.98 ^a^	24.45±2.2	21.52±3.1	23.14± 2.3
36 Months	-	29.69±5.1	-	26.16±4.6	*	*

*; dead trees

a; only one replicate was PCR positive. Standard errors were calculated from three replicates.

## Discussion

HLB, a phloem restricted bacterial disease of citrus has been present in the United States since 2005 [33]. This disease has resulted in a severe decline in fruit production in Florida, where it has become endemic [34]. Florida produces sweet oranges, predominantly for juice production and all commercial cultivars are susceptible to HLB [35]. Development of new cultivars through conventional hybridization is very difficult due to the high level of nuclear embryony in these cultivars. All major commercial sweet orange cultivars have arisen through the development of mutations and have been subsequently selected over hundreds of years [36]. In such cases, genetic improvement of existing cultivars without otherwise changing its characteristics through the incorporation of an additional advantageous trait remains the fastest method of improvement. Genetic engineering of sweet oranges is a viable alternative to conventional breeding as it is a relatively rapid process and it allows for the insertion of a single trait without the modifying existing traits. In this study, ‘Hamlin’ and ‘Valencia’ sweet oranges were transformed with the *AtNPR1* transgene via *Agrobacterium-*mediated genetic transformation to produce transgenic plants tolerant to HLB.

NPR1 is a key regulator of gene expression following infection [37] and controls the onset of the immune response known as SAR [38]. *AtNPR1* has been directly implicated for fungal disease resistance in wheat [39], cotton [40], broad spectrum disease resistance in strawberry [41], tomato [42], carrot [43] and bacterial disease resistance in citrus [11]. In addition, *AtNPR1* homologs have been identified in many economically important plants such as citrus [44], gladiolus [45], grapevine [46], rice [47], phalaenopsis orchid [48] and sugarbeet [49] among others. Development of HLB resistant citrus by exploiting the plants own immune system is a potentially attractive approach to develop a genetically modified consumer-acceptable plant. This strategy utilizes a transgene whose homolog is available in many of our food crops, including citrus.

Regenerated trees exhibited normal phenotypes and did not demonstrate the abnormalities that were observed in strawberry plants constitutively expressing *AtNPR1* [41] or the rice *AtNPR1* homolog (NH1) in rice [47]. Homology dependent gene silencing can be an issue when endogenous genes are overexpressed in the same system [50, 51], which led us to overexpress the *AtNPR1* in these citrus plants instead of the citrus homolog. Our results indicate overexpression of the *AtNPR1* gene can induce resistance to HLB with a reduced disease severity phenotype in many lines. Resistance could not be directly co-related to *AtNPR1* gene expression levels as several transgenic lines with good *AtNPR1* expression levels were susceptible to HLB. This could be due to differential insertion of the transgene cassette in the individual lines. *AtNPR1* produced either constitutively or in the phloem was observed to be sufficient in combatting HLB. Since NPR1 regulates the signal transduction pathway that results in SAR [18], gene expression in the phloem cells was sufficient to induce the PR genes resulting in disease resistance. Molecular analyses revealed the presence of the coding sequence of the introduced *AtNPR1* and the expression of the gene in transgenic sweet orange plants. Analyzed lines had less than 3 copies of the transgene stably incorporated into the genome. We had previously observed that increase in copy number negatively affected the gene expression in citrus [52] and current results support that observation. Three genes involved in the plant defense signaling pathways, *PR1* [37, 53], *PR2* [11, 54] and *WRKY70* [55, 56] were evaluated in this study based on their ability to be differentially regulated by *AtNPR1*. *AtNPR1* induces *PR1* gene expression [31] and the single copy insert (transgenic line 2) had both the highest *NPR1* expression and *PR1* expression. In fact, levels of *PR1* gene expression could be directly co-related to the transgene mediated resistance to HLB. *PR2*, which has been observed to be directly responsible for the SAR process [11, 57] was also overexpressed in all the transgenic lines, though not at the levels observed for *PR1* expression. The *WRKY70* transcription factor influences the defense pathways [58] and specifically the salicylate-mediated signaling pathways in plant defense [56]. Apart from line 18, all the other lines behaved similarly demonstrating the activation of the SAR pathways. The observed results are contradictory to that observed before in citrus [11] where constitutive defense responses were not observed following overexpression of *AtNPR1*. A few of the transformed lines did not exhibit enhanced gene expression indicating post-transcriptional gene silencing or inefficient nicking of T-DNA borders and co-transfer of non-T-DNA sequences into the citrus genome[59,60]. The basic mechanism behind SAR is generally conserved across species, but based on our results, it becomes apparent that there is a differential gene expression pattern following SAR between citrus and other crop plants.

## Conclusions

In addition to inducing resistance to HLB, the SAR response observed could potentially protect our trees from other important citrus fungal and bacterial diseases such as citrus canker and black spot. Both constitutive expression and phloem expression of *AtNPR1* would lead to a genetically modified commercial scion and in addition, phloem expression could lead to the development of a transposable transgene effect that could possibly induce HLB resistance in non-transgenic citrus. Phloem specific expression of the transgene and the observed resistance could allow the movement of the SAR response across the graft union. This transfer may induce a SAR response that could potentially protect the non-transgenic scion from HLB. In this model any existing non-transgenic scion could be budded onto a transgenic rootstock in order to impart HLB resistance. A non-transgenic scion grafted onto a transgenic rootstock could potentially be acceptable to the consumer than transgenic citrus scions. In addition, this transgene can also serve as a component for the development of an all plant T-DNA derived consumer friendly GM tree.
