# Correction: Transgenic Citrus Expressing an *Arabidopsis* NPR1 Gene Exhibit Enhanced Resistance against Huanglongbing (HLB; Citrus Greening)

**DOI:** 10.1371/journal.pone.0147657

**Published:** 2016-01-19

**Authors:** Manjul Dutt, Gary Barthe, Michael Irey, Jude Grosser

The authors would like to acknowledge the use of the Bi-directional dual promoter complex with enhanced promoter activity for transgene expression in eukaryotes in this study [2]. The authors would like to add the following citations to the References: Li, Z. and D. J. Gray, Bi-directional dual promoter complex with enhanced promoter activity for transgene expression in eukaryotes, US Patent 7,129,343, 2006 [2], and Li, Z., S. Jayasankar and D. J. Gray, Bi-directional duplex promoters with duplicated enhancers significantly increase transgene expression in grape and tobacco. Trans. Res. 13, 2004, 143–154 [3].
